# The Pneumonia Severity Index as a Predictor of In-Hospital Mortality in Acute Exacerbation of Chronic Obstructive Pulmonary Disease

**DOI:** 10.1371/journal.pone.0133160

**Published:** 2015-07-17

**Authors:** Guoping Hu, Yumin Zhou, Yankui Wu, Yan Yu, Weiqiang Liang, Pixin Ran

**Affiliations:** 1 Department of Respiratory Medicine, The Third Affiliated Hospital of Guangzhou Medical University, Guangzhou, Guangdong, China; 2 Guangzhou Institute of Respiratory Diseases, State Key Lab of Respiratory Diseases, The First Affiliated Hospital, Guangzhou Medical University, Guangzhou, Guangdong, China; 3 Department of Respiratory Disease of People’s Hospital of Guangxi Zhuang Autonomous Region, Guangxi Zhuang Autonomous Region, P R China; University of Western Australia, AUSTRALIA

## Abstract

**Objective:**

To determine whether the pneumonia severity index (PSI) can predict in-hospital mortality for AECOPD patients and compare its usefulness with the CURB65 and BAP65 indexes to predict mortality.

**Methods:**

Demographics, clinical signs and symptoms, comorbidities, and laboratory and radiographic findings of hospitalized AECOPD patients were obtained. Univariate and multiple logistic regression analyses were used to identify the risk factors for in-hospital mortality. The PSI, CURB65 and BAP65 scores were calculated. Receiver operating characteristic (ROC) curve analysis was used to identify the PSI, CURB65 and BAP65 scores that could discriminate between non-survivors and survivors. To control for the confounding factor of invasive mechanical ventilation (IMV) regarding the mortality of AECOPD, subgroup analysis was performed when excluded patients who had met the criteria of IMV but who had not received the cure of IMV according to their wishes.

**Results:**

During the in-hospital period, 73 patients died and 679 patients recovered. Age, PaO_2_<60 mmHg, pH < 7.35, PaCO2≥50 mmHg, nursing home residency, congestive heart failure, liver disease, sodium<130 mmol/L, lower FEV1% and altered mental status were risk factors for in-hospital mortality. The areas under the ROC curves (AUCs) of the PSI for death were 0.847 (95% CI: 0.799-0.895). The cut-off value was 116.5 with a sensitivity of 82.2% and a specificity of 77.6%. However, the AUCs of the CURB65 and BAP65 for death were only 0.744 (95% CI: 0.680-0.809) and 0.665 (95% CI: 0.594-0.736), respectively. Subgroup analysis also showed that the PSI score could predict the mortality of AECOPD patients with an AUC = 0.857 (95% CI: 0.802-0.913), with exclusion of the patients who met the criteria of IMV but who did not receive the cure of IMV.

**Conclusion:**

The PSI score may be used to predict in-hospital mortality for hospitalized AECOPD patients, with a prognostic capacity superior to CURB65 and BAP65.

## Introduction

Episodes of an acute exacerbation of chronic obstructive pulmonary disease (AECOPD) are the main cause of disease-related costs, morbidity, and mortality[1]. AECOPD is also the third leading cause of death in the world[2]. Therefore, tools that can reliably identify patients who are in the terminal stages of the disease are clinically desirable[3].

A risk marker that reflects the real-life clinical situation and identifies mortality risk in AECOPD patients is clinically desirable. Such a marker could be used to triage patients who require hospitalization versus those patients who require a lower level of health care[4]. An effective risk marker would also determine those in the high-risk group who require more intensive monitoring and care. With the exception of lung function decline, previous studies have reported several prognostic markers of COPD [5] [6–13]. In the setting of acute exacerbations, studies have also shown the prognostic value of COPD that included the frequency of exacerbations, hypercapnia and serum uric acid [13–22]. COPD patients have an increased prevalence of cancer, cardiovascular disease and depression compared with the general population[23]. Prospective studies have examined COPD comorbidities and mortality risk[24]. The meta-analysis by Aran[25] reported that twelve prognostic factors (age, male sex, low body mass index, cardiac failure, chronic renal failure, confusion, long-term oxygen therapy, lower limb edema, Global Initiative for Chronic Lung Disease criteria stage 4, corpulmonale, acidemia, and an elevated plasma troponin level) were significantly associated with increased short-term mortality, indicating that these parameters may be useful to develop tools for the prediction of outcome in clinical practice. However, most of the studies that assessed the predictive role of markers contained too many exclusion criteria that do not reflect real life, thus limiting the usefulness of these markers. Additionally, most of the factors had been validated in only one study with no independent validation[25].

CURB65 (confusion, urea > 7 mmol/L, respiratory rate>30/min, blood pressure systolic < 90 mm Hg and age > 65 years) and BAP65 (urea, confusion, heart rate, age > 65 years) were the most frequently studied scores[26–29]. However, the predictive value of existing scores was modest (area under the curve, 0.7–0.8), suggesting that more accurate prediction tools are needed[30].

The PSI prediction rule assigns points based on age, comorbidities, abnormal physical findings (such as a pulse ≥125/min or systolic blood pressure <90 mm Hg) and abnormal laboratory findings (such as a hematocrit <30%, partial pressure of arterial oxygen <60 mm Hg or blood glucose level ≥250 mg/dl (14 mmol/liter)) at presentation[31]. Yoon K Loke and colleagues[32] performed a meta-analysis to determine the ability of PSI to correctly predict mortality in patients with pneumonia, and showed that the PSI performed well at identifying patients with pneumonia who had a low risk of death[32]. Another system review[33] also showed that PSI could predict the 30 day mortality of CAP, with an area under the sROC curve of 0.8.

The PSI score reflects more comprehensively the real-life clinical situation, and it is an effective prognostic predictor for CAP. There is often considerable overlap in the clinical presentation of COPD exacerbation and pneumonia[28]. Additionally, many of the variables in the PSI system have been proven to be prognostic factors for AECOPD. However, to date, no study has assessed the prognostic value of the PSI score regarding the admission of AECOPD patients. Therefore, we investigated whether the PSI score could effectively predict in-hospital mortality in AECOPD patients and compared its usefulness with the CURB65 and BAP65 indexes to predict mortality.

## Methods

### Study design

The study was prospectively conducted at the third Affiliated Hospital of Guangzhou Medical University, Guangzhou, Guangdong, China, from July 2010 to May 2014. The diagnosis of AECOPD was supported by spirometric evidence of airflow obstruction (forced expiratory volume in one second (FEV1)/forced vital capacity (FVC) < 0.70) when clinically stable[1]. Exacerbations were defined as dyspnea, cough or sputum purulence severe enough to warrant hospitalization[1] that were diagnosed by the admitting physicians. The inclusion criterion was COPD exacerbation requiring hospitalization. The exclusion criteria were hospitalization for a reason other than AECOPD, a history of other respiratory illnesses such as penumoconiosis or interstitial lung disease,and AECOPD be triggered by pneumonia according to chest radiography. This study was observational, and treatment was given according to the patient's condition, which was not influenced by participation in the study. When the patients had the indication for noninvasive mechanical ventilation (NIV)[1], it was administered to the patients. Additionally, when there is a need for invasive mechanical ventilation (IMV)[1], it was administered to the patients according to their wishes, and a clear statement of the patients’ wishes was obtained. The primary end-point was in-hospital mortality. The study was conducted according to the principles of the Declaration of Helsinki. The research protocols were approved by the ethics committee of the Third Affiliated Hospital of Guangzhou Medical University. Written informed consent was obtained from all of the participating patients.

### Data collection

A questionnaire including the demographic data, comorbidities (history of the tumor, heart failure, liver disease and renal dysfunction) and physical examination (mental status, respiratory rate, pulse, blood pressure and body temperature) was filled out by a respiratory medicine resident. Arterial blood gas analysis (partial pressure of oxygen, partial pressure of carbon dioxide (PCO_2_) and pH), as well as blood urea nitrogen, sodium, blood glucose and hematocrit levels were measured, within 4 h of admission. Confusion was assessed by the admitting clinician. The PSI scores were calculated using 20 items [31]. The prediction rule assigns points based on age, comorbidities, laboratory tests and abnormal physical findings[31].

### Statistical analysis

Continuous variables were shown as the mean ± standard deviation. Categorical variables were presented as absolute numbers and proportions. Univariate analysis and logistic regression analysis were used to identify independent variables associated with death. Differences between the groups were compared by chi-squared test for categorical variables and one-way analysis of variance for continuous variables. To limit the number of events-per-variable, variables with a p-value <0.2 in univariate analysis were included in the logistic regression model[34]. A receiver operating curve (ROC) for predicting in-hospital mortality was calculated. To control the confounding factor of IMV for the mortality of AECOPD, a subgroup analysis was performed to evaluate the value of PSI for the in-hospital mortality of AECOPD, with exclusion of the patients for whom IMV was not administered according to the patients wishes but who had met the criteria of IMV. The data were analyzed using a Stata statistical software package (Version 7.0; Stata Corporation, College Station, TX, USA). A two-sided p-value <0.05 was considered statistically significant.

## Results

### Baseline characteristics

Table 1 summarizes the baseline characteristics of the study population. There were 752 patients with AECOPD included in our cohort study. Overall, 132 patients (17.6%) had congestive heart failure, 128 (17.0%) had renal disease, 36 (4.8%) had liver disease, and 108 (14.4%) had cerebrovascular disease during their hospital stay. No patient had pleural effusion and a temperature <35°C or >40°C. A comparison between the survivors and deceased hospitalized patients is presented in Table 1. The patients who died were more likely to have renal disease, heart failure and liver disease. In addition, the patients who died were significantly more hypercapnic (PaCO_2_: 9.82±3.96 kpa) and older (80.53±9.36 years old) than the survivors (PaCO2: 6.38±2.36 kpa and 77.56±8.82 years old). The pH, PaO_2_, hematocrit and sodium levels were significantly lower in the non-survivors (pH: 7.26±0.16, PO_2_: 10.08±4.41 kpa, hematocrit: 35.58±7.27, sodium: 138.01±7.50 mmol/l) than in the survivors (pH: 7.38±0.06, PO_2_: 11.27±3.47 kpa, hematocrit: 38.08±5.67, sodium: 139.72±4.15 mmol/l). The plasma concentrations of blood urea nitrogen, respiratory rate and heart rate were higher in the non-survivors (BUN: 11.28±10.12 mmol/L, respiratory rate: 24.5±5.5/min, heart rate: 100.7±20.6/min) than in the survivors (BUN: 6.76±3.82 mmol/L, respiratory rate: 22.1±2.7/min, heart rate: 94.6±16.7/min). There were 49 patients who met the criteria of IMV; however, according to the patients’ wishes, there were only 24 patients who were treated by invasive mechanical ventilation. There were 38 patients who were treated by noninvasive mechanical ventilation. There were 17,9, and 19 patients died among those who were supported by noninvasive mechanical ventilation, invasive mechanical ventilation and those who met the IMV criteria but did not receive IMV.

**Table 1:** Baseline characteristics and survival of patients hospitalized with AECOPD.

Patient Characteristics	Total (752)	Alive (679)	Deceased (73)	X2/F	P
Age (yrs)	77.85±8.91	77.56±8.82	80.53±9.36	7.421	0.007
Gender (male/female)	212/540	193/486	19/54	0.187	0.784
Smoker (no/yes)	129/623	116/563	13/60	0.02	0.876
NHR (yes/no)	32/720	19/660	13/60	36.449	0.000
PaCO_2_ (kpa)	6.71±2.45	6.38±2.36	9.82±3.96	780.82	0.000
PaO_2_ (kpa)	11.15±3.58	11.27±3.47	10.08±4.41	7.348	0.007
pH	7.37±0.08	7.38±0.06	7.26±0.16	168.458	0.000
FEV1/FVC	53.78±7.32	53.62±7.22	55.29±8.10	3.472	0.063
FEV1%	56.1±19.4	56.6±19.4	51.9±19.1	3.871	0.049
Neoplastic disease	73/679	65/614	8/65	0.144	0.678
CHF	132/620	96/583	36/37	56.358	0.000
Renal disease	128/624	103/576	25/48	16.984	0.000
Liver disease	36/716	26/653	10/63	14.086	0.001
Heart rate	95.2±17.2	94.6±16.7	100.7±20.6	8.183	0.004
Respiratory rate	22.3±3.2	22.1±2.7	24.5±5.5	40.607	0.000
SBP	134.9±21.5	136.0±20.5	123.6±27.5	20.591	0.000
Altered mental status	35/717	20/659	15/58	46.022	0.000
Blood urea nitrogen	7.20±4.98	6.76±3.82	11.28±10.12	58.450	0.000
Sodium	139.56±4.61	139.72±4.2	138.01±7.5	9.217	0.002
Glucose	5.85±2.39	5.76±2.15	6.69±3.92	10.057	0.002
Hematocrit	37.83±5.88	38.08±5.67	35.58±7.27	12.043	0.001
Albumin	36.36±5.00	36.86±4.60	31.66±5.71	80.171	0.000
Cerebrovascular disease (yes/no)	108/644	90/589	18/55	6.968	0.008
NIV (yes/no)	38/714	21/658	17/56	56.029	0.000
IMV (yes/no)	24/728	15/664	9/64	21.847	0.000

NHR: nursing home resident, PaO_2_: partial pressure of arterial oxygen; PaCO_2_: partial pressure of arterial carbon dioxide. FEV1%, predicted forced expiratory volume in one second; FEV1/FVC, forced expiratory volume in one second/forced vital capacity; CHF: congestive heart failure; SBP: systolic blood pressure; NIV: noninvasive mechanical ventilation; IMV: invasive mechanical ventilation.

### Risk factors for AECOPD

Univariate analyses (Table 2) and logistic regression analysis (Table 3) confirmed that a pH less than 7.35, PaCO_2_ not less than 50 mmHg, PaO_2_ less than 60 mmHg, nursing home residency status, congestive heart failure, liver disease, sodium<130 mmol/L, lower FEV1%, and altered mental status were risk factors of in-hospital death. Using univariate analysis, older age was not a risk factor for in-hospital death. However, logistic regression analysis confirmed that older age was a risk factor for in-hospital death.

**Table 2:** Mortality Risk in Patients with AECOPD.

Characteristics	*Alive N* (%)	*Death N* (%)	*RR*	X^2^	P
**Age (yrs)**				7.781	0.100
-59 yrs	26	2	1		
60–69 yrs	98	5	0.68 (0.14–3.32)		
70–79 yrs	219	18	1.06 (0.26–4.34)		
80–89 yrs	287	40	1.71 (0.44–6.72)		
≥90 yrs	8	49	12.04 (3.15–45.94)		
**Smoking**				0.02	0.876
No	116 (89.9%)	13 (10.1%)	1		
Yes	563 (90.4%)	60 (9.6%)	0.96 (0.54–1.69)		
**Sex**				0.19	0.665
Male	193 (91%)	19 (9%)	1		
Female	486 (88.3%)	54 (11.7%)	1.12 (0.68–1.84)		
**Nursing home resident**				36.45	0.000
No	660 (94.8%)	60 (5.2%)	1		
Yes	19 (71.6%)	13 (28.4%)	4.88 (3.00–4.91)		
**Neoplastic disease**				0.14	0.070
No	614 (90.4%)	65 (9.6%)	1		
Yes	65 (89%)	8 (11%)	1.14 (0.57–2.29)		
**Liver disease**				14.09	0.002
No	653 (91.2%)	63 (8.8%)	1		
Yes	26 (72.2%)	10 (27.8%)	3.16 (1.77–5.62)		
**Congestive heart failure**				56.36	0.000
No	583 (94%)	37 (6%)	1		
Yes	96 (72.7%)	36 (27.3%)	4.57 (3.01–6.95)	6.97	0.083
**Cerebrovascular disease**					
No	589	55	1		
Yes	90	18	2.14 (1.13–3.90)		
**Renal disease**				16.98	0.000
No	576 (90.1%)	48 (7.7%)	1		
Yes	103 (77.4%)	25 (19.5%)	2.54 (1.63–3.96)		
**Altered mental status**				46.02	0.000
No	659 (91.9%)	58 (8.1%)	1		
Yes	20 (57.1%)	15 (42.9%)	5.30 (3.36–8.35)		
**Pulse ≥125/min**				11.91	0.001
No	650 (91.2%)	63 (8.8%)	1		
Yes	29 (74.4%)	10 (25.6%)	2.90 (1.62–5.20)		
**Respiratory rate**				29.03	0.000
<30/min	662 (91.4%)	62 (8.6%)	1		
≥30/min	17 (60.7%)	11 (39.3%)	4.59 (2.73–7.70)		
**Systolic blood pressure**				55.59	0.000
≥90 mmHg	672 (91.6)	62 (8.4%)	1		
<90 mmHg	7 (8.3%)	11 (91.7%)	17.03 (5.74–53.33)		
**Blood Urea nitrogen**				18.74	0.000
<11 mmol/L	555 (92.7%)	44 (7.3%)	1		
≥11 mmol/L	124 (81%)	29 (19%)	2.58 (1.67–3.98)		
**Sodium**				30.87	0.000
≥130 mmol/L	666 (91.4)	63 (8.6%)	1		
<130 mmol/L	13 (56.5%)	10 (43.5%)	5.03 (2.98–8.48)		
**Glucose**				2.70	0.100
<14 mmol/L	669 (90.5)	70 (9.5%)	1		
≥14 mmol/L	10 (76.9%)	3 (23.1%)	2.44 (0.88–6.74)		
**Hematocrit**				3.70	0.073
≥30%	634 (90.8)	64 (9.2%)	1		
<30%	45 (83.3%)	9 (16.7%)	1.82 (0.96–3.45)		
**Arterial pH**				158.38	0.000
≥7.35	523 (95.1)	27 (4.9%)	1		
7.20–7.35	147 (86.5%)	23 (13.5%)	3.03 (1.61–5.66)		
-7.20	9 (28.1%)	23 (71.9%)	49.50 (19.5–131.4)		
**PaO**_**2**_				55.85	0.000
≥60 mmHg	597 (93.7%)	40 (6.3%)	1		
<60 mmHg	82 (71.3%)	33 (28.7%)	6.0 (3.45–10.35)		
**PaCO**_**2**_				59.40	0.000
<50 mmHg	474 (96.3%)	18 (3.7%)	1		
≥50 mmHg	205 (78.8%)	55 (21.2%)	7.06 (3.96–13.08)		
**FEV1%**				59.40	0.000
≥80%	84 (96.3%)	3 (3.7%)	1		
50–80%	336 (92.6%)	27 (7.4%)	2.16 (0.67–6.9)		
30–50%	199 (90.9%)	20 (9.1%)	2.64 (0.81–8.69)		
-30%	60 (72.3%)	23 (27.7%)	8.03 (2.51–25.7)		

PaO_2_: partial pressure of arterial oxygen; PaCO_2_: partial pressure of arterial carbon dioxide; FEV1%, predicted forced expiratory volume in one second.

**Table 3:** Logistic regression analyses of the risk factors associated with mortality in AECOPD patients.

	B	SE	Wald	P	Exp (B)	95% CI for EXP (B)	
						lower	Upper
Liver disease	1.498	0.581	6.645	0.010	4.471	1.432	13.962
PaCO_2_	1.039	0.411	6.380	0.012	2.826	1.262	6.329
Nursing home resident	1.745	0.555	9.886	0.002	5.725	1.929	16.985
PaO_2_	1.078	0.365	8.732	0.003	2.938	1.437	6.004
pH	0.986	0.403	5.973	0.015	2.680	1.216	5.908
confusion	1.249	0.504	6.143	0.013	3.488	1.299	9.369
sodium	1.427	0.673	4.489	0.034	4.165	1.113	15.591
CHF	1.545	0.351	19.425	0.000	4.689	2.359	9.323
FEV1%	0.802	0.210	14.576	0.000	2.230	1.477	3.366
Age	0.437	0.203	4.653	0.031	1.548	1.041	2.303
Constant	-7.238	0.771	88.076	0.00	0.001		

CHF: congestive heart failure; PaO_2_: partial pressure of arterial oxygen; PaCO_2_: partial pressure of arterial carbon dioxide.

### ROC curves of PSI, CURB65, BAP65 for AECOPD death

**Fig 1:**
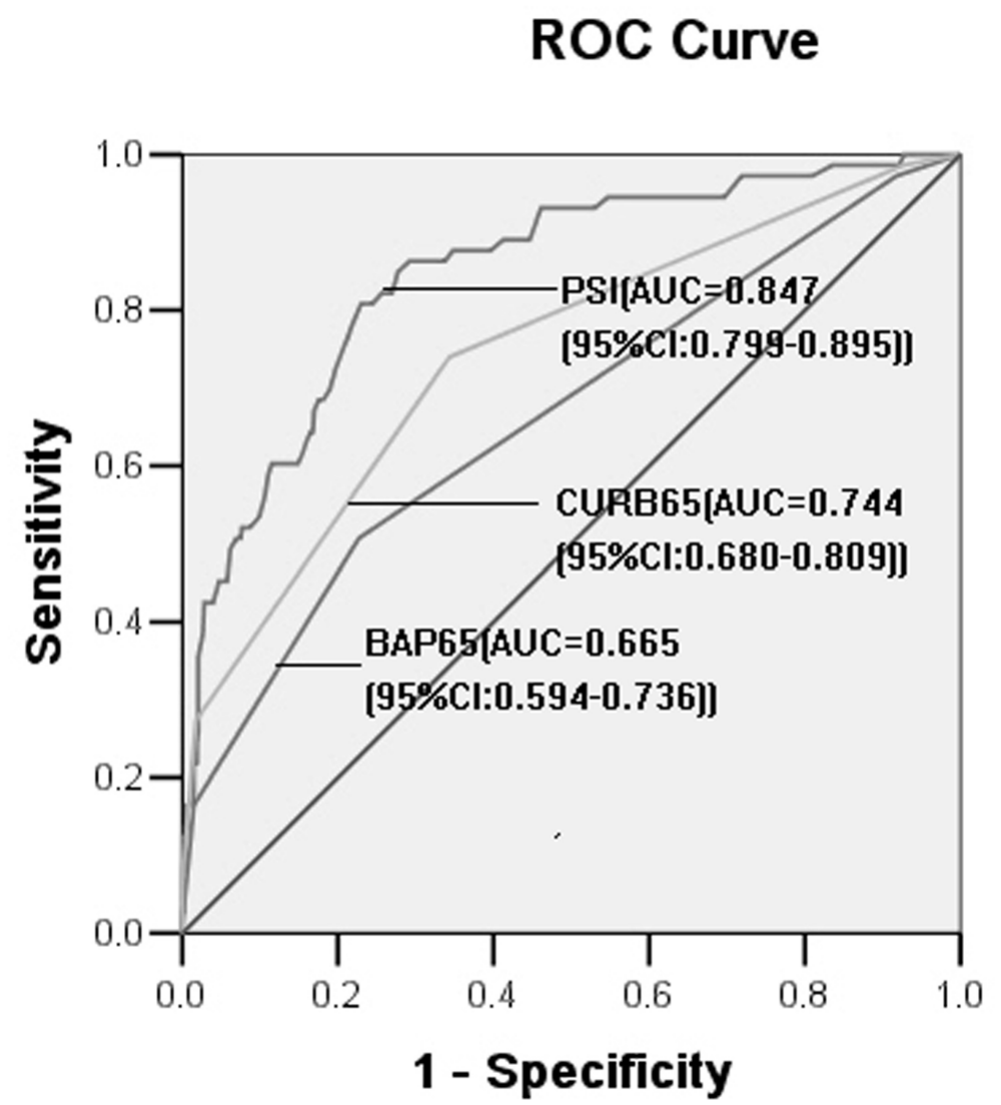
Receiver operating characteristic (ROC) curves for the PSI, CURB65 and BAP65 scores to predict in-hospital mortality in AECOPD patients. AUC: area under the ROC curve.

In-hospital mortality according to the PSI, CURB65 and BAP65 score is shown in Table 4. In-hospital mortality increased with the PSI, CURB65 and BAP65 score. ROC curves were used to determine the cut-off values for the PSI, CURB65 and BAP65 scores. The optimal values of the PSI, CURB65 and BAP65 scores for predicting death were defined as the PSI, CURB65 and BAP65 scores with the largest sensitivity plus specificity for the curves. The PSI score had good discriminative capability for death with AUC of 0.847 (95% confidence interval (CI) = 0.799–0.895, *P* = 0.000), displaying good internal validity. A total score of 116.5 had a sensitivity of 82.2% and a specificity of 77.6% (Fig 1).

**Table 4:** In-hospital mortality according to the PSI, CURB65 and BAP65 score and risk groups for AECOPD.

Characteristics	*Alive N* (%)	*Deceased N* (%)	*OR*	X^2^	P
**PSI**					
-70	83 (100%)	0 (0%)			
71–90	235 (98.7%)	3 (1.3%)	1		
91–130	272 (91.3%)	26 (8.7%)	7.49 (2.25–39.03)	14.41	<0.01
>130	89 (66.9%)	44 (33.1%)	38.73(11.8–197.9)	78.09	<0.01
**PSI**				108.0	<0.01
<116.5	527 (97.4%)	13 (2.4%)	1		
>116.5	152 (72.6%)	60 (28.3%)	16.0 (8.37–32.51)		
**CURB65**					
0	50 (98%)	1 (2%)	1		
1	396 (95.7%)	18 (4.3%)	2.27 (0.34–96.54)	0.66	0.417
2	221 (91.3%)	34 (13.3%)	7.69 (1.22–318.75)	5.43	0.020
3–5	12 (37.5%)	20 (62.5%)	83.33(10.5–3518.8)	38.13	<0.01
**BAP65**					
0	56 (96.6%)	2 (3.4%)	1		
1	469 (93.2%)	34 (6.8%)	2.03 (0.50–17.88)	0.95	0.330
2	144 (85.2%)	25 (14.8%)	4.86 (1.14–43.50)	5.30	0.021
3–4	10 (45.5%)	12 (54.5%)	33.6 (5.79–330.06)	28.84	<0.01

PSI: pneumonia severity index.

The CURB65 and BAP65 scores had some discriminative capability for death with AUCs of 0.744 (95% CI = 0.680–0.809, *P* = 0.000) and 0.665 (95% CI = 0.594–0.736, *P* = 0.000), respectively (Fig 1), indicating that PSI has a prognostic capacity superior to that of CURB65 and BAP65.

**Fig 2:**
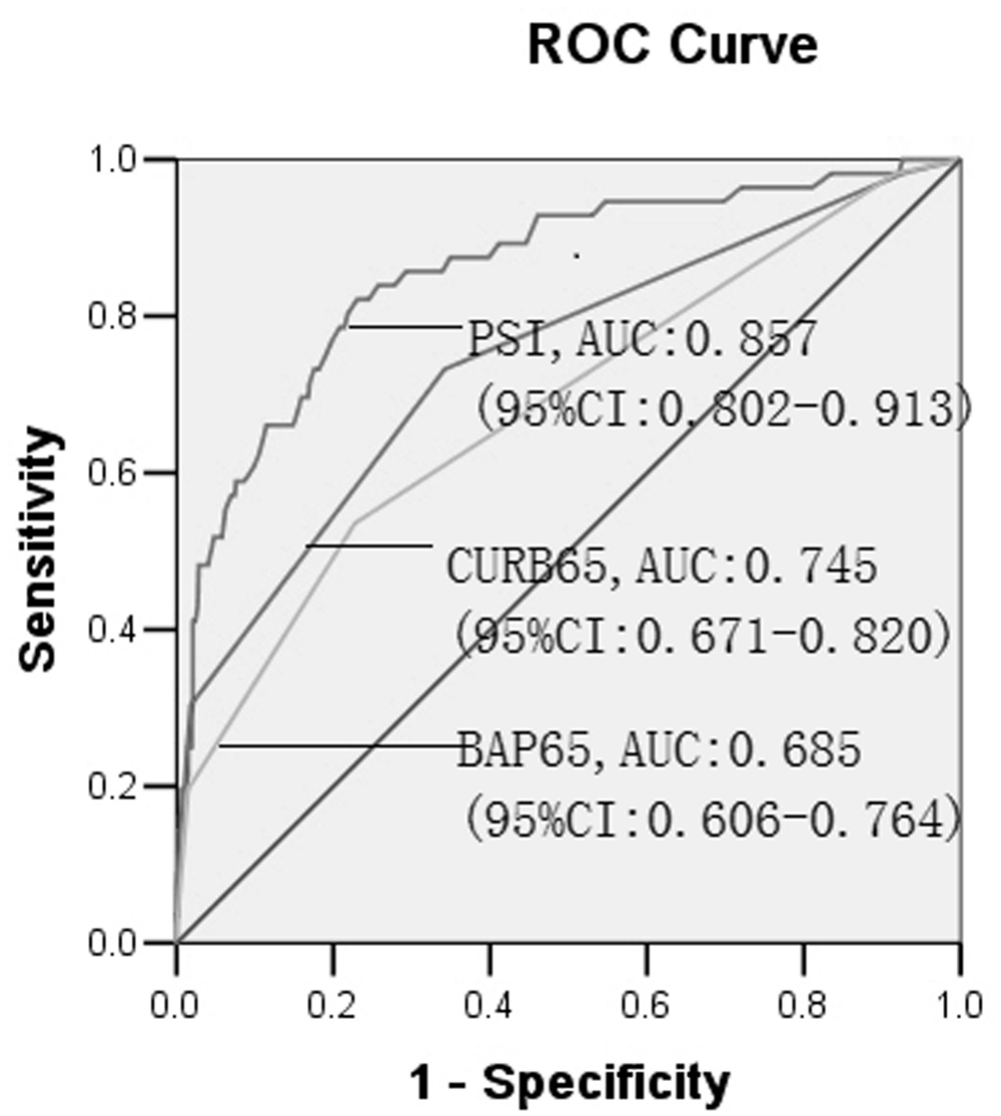
Receiver operating characteristic (ROC) curves for PSI, CURB65 and BAP65 score to predict in-hospital mortality in AECOPD patients with the exclusion of patients who met the criteria of IMV but who did not receive IMV according to the patients’ wishes. AUC: area under the ROC curve; IMV: invasive mechanical ventilation.

To control for the confounding factor of IMV regarding the mortality of AECOPD, we performed subgroup analysis to evaluate the discriminative capability of PSI, CURB65 and BAP65 for death in AECOPD. When the patients who met the criteria of IMV but who did not receive the cure of IMV were excluded, the PSI score also had good discriminative capability for death with an AUC = 0.857 (95% CI: 0.802–0.913, *P* = 0.000). A total score of 116.5 had a sensitivity of 82.1% and a specificity of 77.0% (Fig 2). The CURB65 and BAP65 scores also had some discriminative capability for death with AUCs of 0.745 (95% CI: 0.671–0.820, *P* = 0.000) and 0.685 (95% CI = 0.606–0.764, *P* = 0.000), respectively (Fig 2), also indicating that PSI has a prognostic capacity superior to that of CURB65 and BAP65.

### Mortality for the PSI and AECOPD scores

The entire cohort was divided into two groups according to the PSI score. There were 212 patients with a PSI score ≥116.5 and 540 patients with a PSI score <116.5. The in-hospital mortality for the PSI, CURB65, and BAP65 scores are shown in Tables 4 and 5, which show that the mortality increased with increasing PSI, CURB65, and BAP65 score. Compared with a PSI score <116.5, a PSI score ≥116.5 was an in-hospital mortality predictor (OR = 16.0, p < 0.05). The risk of in-hospital mortality increased with higher PSI scores.

**Table 5:** Comparison of in-hospital mortality according to the PSI risk group in COPD exacerbation and community-acquired pneumonia.

	no. of deceased AECOPD patients	no. of patients	% that died of AECOPD	% that died of CAP[31]
II (≤70)	0	83	0%	0.6%
III (71–90)	3	238	1.3%	0.9%
IV (91–130)	26	298	8.7%	9.3%
V (>130)	44	133	33.1%	27%
TOTAL			9.7%	5.2%

Table 6 shows the subgroup analysis when the patients who met the criteria of IMV but who did not receive IMV were excluded. The subgroup analysis also showed the mortality increased with increasing PSI, CURB65, and BAP65 score.

**Table 6:** Sub-group analysis of in-hospital mortality by the PSI, CURB65 and BAP65 score and risk groups for AECOPD when the patients who had met the criteria but did not receive the cure of IMV were excluded.

Characteristics	*AliveN* (%)	*DeceasedN* (%)	*OR*	X^2^	P
**PSI**					
-70	83 (100%)	0 (0%)			
71–90	232 (98.7%)	2 (0.9%)	1		
91–130	270 (94.1%)	17 (5.9%)	7.30 (1.70–65.65)	9.42	<0.01
>130	88 (69.2%)	37 (30.8%)	48.77 (12.02–421.91)	69.52.	<0.01
**PSI**				88.79	<0.01
<116.5	522 (98.1%)	10 (1.9%)	1		
>116.5	151 (77.4%)	44 (22.6%)	15.21 (7.29–34.56)		
**CURB65**					
0	50 (98%)	1 (2%)	1		
1	393 (96.6%)	14 (3.4%)	1.78 (0.26–76.79)	0.31	0.576
2	218 (90.1%)	24 (9.9%)	5.50 (0.86–230.78)	3.42	0.064
3–5	12 (41.4%)	17 (58.6%)	70.83 (8.82–3015.79)	34.04	<0.01
**BAP65**					
0	56 (98.2%)	1 (1.8%)	1		
1	464 (94.9%)	25 (5.1%)	3.02 (0.47–126.04)	1.27	0.259
2	143 (88.3%)	19 (11.7%)	7.44 (1.12–314.53)	5.05	0.025
3–4	10 (47.6%)	11 (52.4%)	61.6 (7.01–2695.28)	30.21	<0.01

PSI: pneumonia severity index.

## Discussion

To our knowledge, the present study is the first to prospectively assess the usefulness of PSI scores in AECOPD patients and demonstrated that the PSI score obtained upon hospital admission was an in-hospital mortality predictor in these patients. This study revealed that the in-hospital mortality was 9.7%. The risk of in-hospital mortality was significantly different between the groups and increased with higher PSI scores. A PSI score >116.5 was a strong predictor of in-hospital mortality (OR = 16, 95%CI: 8.37–32.51).

Our study showed that the PSI score had good discriminative capability for in-hospital death with an AUC of 0.860 (95% confidence interval (CI) = 0.816–0.903, *P* = 0.000). Our study also showed that CURB65 and BAP65 could discriminate in-hospital death with AUCs of 0.744 and 0.665, respectively. The latter finding indicates that CURB65 and BAP65 were less effective than PSI in predicting in-hospital mortality for AECOPD. Many studies have been performed to predict the mortality of AECOPD. As reported by Aran [30], 10 studies evaluated severity prediction tools specifically for outcome in COPD exacerbation. The area under the curve for these scores ranged from 0.68 for CRB-65 to 0.83 for a derived score by Roche and colleagues[4, 35]. Until now, the predictive value of the reported prediction tools was modest in predictive mortality, while the PSI score was a more robust predictor of mortality with more accurate prediction.

The PSI score could distinguish AECOPD patients with a high risk of short-term mortality from those in the low-risk group. According to the PSI score, the low-risk group may be considered for treatment at home, whereas the high-risk group may be require intensive hospital care. Our study demonstrated that the PSI score was an effective mortality predictor for acute COPD exacerbation, as was shown in previous cohorts with CAP[31].

The most common comorbidities observed in our study were congestive heart failure (17.55%), renal disease (17.02%), liver disease (5.05%), and cerebrovascular disease (14.36%). Mortality was also higher in the patients with congestive heart failure.

The association between COPD and cardiovascular disease is well documented in the literature and is a common cause of mortality in these patients[24, 36].

Research has reported that the presence of preexisting comorbidities and corpulmonale was associated with reduced survival for AECOPD patients[1]. Even in studies in which patients with severe comorbidities were excluded by design, a significant percentage of patients died of cardiovascular disease or cancer during the follow-up[29, 37]. Some studies have explored the prognostic markers of COPD[24, 29, 36–41], among which the CODEX index is useful in predicting survival and hospital readmissions at both 3 months and 1 year after hospital discharge for a COPD exacerbation[36]. However, because only a single study shows good performance characteristics of a score does not mean that this information will be useful or should be used in clinical practice. Some studies have reported that tools (such as CODEX[36], DOSE[42], and updated ADO indexes[43]) to predict mortality, but most of them have not been externally validated and implemented in clinical practice. One explanation was that there were too many inclusion and exclusion criteria. Another explanation was that the predictive value of existing scores was a modest predictor, not a strong predictor. A third explanation was that the tools included some subjective variable (for example, dyspnea was a subjective feeling).

Information from a marker that can predict the mortality for AECOPD would have several advantages. Although it may not improve the patient outcome, this information may facilitate discussions between clinicians, patients and their families and may be helpful for making management decisions. For service providers, they can assess the needs of different patient populations according to their severity information.

Our study has several limitations. First, we only assessed the role of the PSI score in in-hospital mortality for AECOPD; we did not explore the effect of the PSI score on the long-term mortality of COPD. Second, COPD includes many types of coexisting diseases, and some were not included in the PSI score, which may bias the effect of PSI on AECOPD mortality. Third, as a shortcoming of our study, we did not collect information concerning the history of exacerbation; thus, we cannot provide the information regarding exacerbation history because a study by Celli showed that a history of exacerbation was the best predictor of AECOPD [5], which could cause some bias. The fourth limitation was the sample selection. We only included inpatients admitted to the respiratory department, and these patients were thus more likely to be severely ill and aged. Our results were only from a single center with a modest number of patients; thus, to generalize our findings, further confirmation with larger numbers of patients and at other centers would be required.

## Conclusion

In summary, the PSI score could predict in-hospital mortality for AECOPD patients requiring admission to the hospital, with a prognostic capacity superior to that of CURB65 and BAP65.

## Supporting Information

S1 Table Data of the patients in the study. NHR: nursing home resident; PaO_2_: partial pressure of arterial oxygen; PaCO_2_: partial pressure of arterial carbon dioxide; FEV1%: predicted forced expiratory volume in one second; FEV1/FVC:forced expiratory volume in one second/forced vital capacity; CHF: congestive heart failure; SBP: systolic blood pressure; NIV: noninvasive mechanical ventilation; IMV: invasive mechanical ventilation; PSI: pneumonia severity index. NRIMVW:not received invasive mechanical ventilation according to the patient wish. Gender: 1 represent female,and 0 represeant male. For the the following item: Liver disease, CHF, Neoplastic disease, Cerebrovascular disease, Renal disease,NHR, Altered mental stutas, NIV, IMV,1 represent yes,0 represent no. NRIMVW:1 represent patient who have not received invasive mechanical ventilation according to the patient wish,0 represent other conditions. Outcome:1 meant death, 0 meant alive.
(XLSX)
